# Permissive weight bearing in trauma patients with fracture of the lower extremities: prospective multicenter comparative cohort study

**DOI:** 10.1186/s12893-018-0341-3

**Published:** 2018-02-02

**Authors:** Pishtiwan H. S. Kalmet, Guido Meys, Yvette Y. v. Horn, Silvia M. A. A. Evers, Henk A. M. Seelen, Paul Hustinx, Heinrich Janzing, Alexander vd Veen, Coen Jaspars, Jan Bernard Sintenie, Taco J. Blokhuis, Martijn Poeze, Peter R. G. Brink

**Affiliations:** 1 Department of Traumatology, Maastricht University Medical Center, P. Debyelaan 25, 6229 HX Maastricht, The Netherlands; 2 Adelante Rehabilitation Center, Hoensbroek, The Netherlands; 3 Maastricht University, School for Public Health and Primary Care: CAPHRI, Maastricht, The Netherlands; 4 Zuyderland Medical Center, Heerlen, The Netherlands; 5 Viecuri Medical Center, Venlo, The Netherlands; 6 Catharina Hospital, Eindhoven, The Netherlands; 7 Maxima Medical Center, Veldhoven, The Netherlands; 8 Elkerliek Hospital, Helmond, The Netherlands

**Keywords:** Fractures of the lower extremities, Trauma patients, Complications, Permissive weight bearing

## Abstract

**Background:**

The standard aftercare treatment in surgically treated trauma patients with fractures around or in a joint, known as (peri)- or intra-articular fractures of the lower extremities, is either non-weight bearing or partial weight bearing. We have developed an early permissive weight bearing post-surgery rehabilitation protocol in surgically treated patients with fractures of the lower extremities. In this proposal we want to compare our early permissive weight bearing protocol to the existing current non-weight bearing guidelines in a prospective comparative cohort study.

**Methods/design:**

The study is a prospective multicenter comparative cohort study in which two rehabilitation aftercare treatments will be contrasted, i.e. permissive weight bearing and non-weight bearing according to the AO-guideline. The study population consists of patients with a surgically treated fracture of the pelvis/acetabulum or a surgically treated (peri)- or intra-articular fracture of the lower extremities. The inclusion period is 12 months. The duration of follow up is 6 months, with measurements taken at baseline, 2,6,12 and 26 weeks post-surgery.

Primary outcome measure: ADL with Lower Extremity Functional Scale. Outcome variables for compliance, as measured with an insole pressure measurement system, encompass peak load and step duration.

**Discussion:**

This study will investigate the (cost-) effectiveness of a permissive weight bearing aftercare protocol. The results will provide evidence whether a permissive weight bearing protocol is more effective than the current non-weight bearing protocol.

**Trial registration:**

The study is registered in the Dutch Trial Register (NTR6077). Date of registration: 01–09-2016.

## Background

The development of surgical fracture care boosted 50 years ago and is improving since, while emphasis on post-surgical care facilitating optimal bone healing and functional recovery remains low [1, 2]. The positive effects of early weight bearing, both for fracture healing and for maintaining muscle and bone mass, are well known. However, little is known about the association between a) the amount or timing of weight bearing and b) bony consolidation or functional recovery. As a result, weight bearing rehabilitation is often cautious and led by existing dogmas, such as the fear for secondary dislocation of the fracture or failure of a mechanical construct. The standard aftercare treatment in surgically treated trauma patients with fractures around or in a joint, known as (peri)- or intra-articular fractures of the lower extremities, is either non-weight bearing or partial weight bearing [3]. According to the AO Principles of Fracture Management, postoperative management of (peri)- or intra-articular fractures of the lower extremities consists of non-weight bearing for 6–12 weeks, followed by partial weight bearing with a 25% increase in weight loading every week [1]. However, in a study by van der Vusse et al. [4] among 111 trauma surgeons and orthopaedic surgeons in the Netherlands, it was shown that consensus about the weight bearing aftercare for tibial plateau fractures is limited. Furthermore, while instructions on rehabilitation provided to patients may be clear, patients’ compliance with a non-weight bearing or limited weight bearing regime has been found to be poor [5, 6]. A number of studies found that patients had actually exceeded the prescribed amount of partial weight bearing even though their self-reported compliance was high [6]. Thus, despite their willingness to comply, patients often do not adhere to the suggested restrictions on weight bearing and increase their weight bearing as fracture healing progresses.

We have developed an early permissive weight bearing post-surgery rehabilitation protocol, where progression of weight bearing is guided by the subjective experience (e.g. pain, weight bearing tolerance) of the patient and therapist, and objective parameters (e.g. temperature of the limb, edema, and gait parameters) are registered. This early permissive weight bearing protocol has previously been implemented and validated in our rehabilitation center since 2005. Follow up and evaluation of the permissive weight bearing protocol are ensured by recording and documenting weight bearing milestones (e.g. walking with 2 crutches, walking with 2 canes, walking with one cane and walking without any walking aids) in a database. Retrospective analysis of the complications that occurred while using this new protocol showed a complication rate of 10%, non-unions and infections being the most common complications. A comparison of our complication rate to data reported in literature that were based on protocols using the existing guidelines, such as the current non-weight bearing guidelines (AO protocol), showed lower complication rates for all our groups treated according to the permissive weight bearing protocol. Recent literature has reported composite postoperative complication rates of up to 37%, with an average of 10–20% in patients with lower extremity fractures [7–16].

In our study we want to compare our early permissive weight bearing protocol to the currently existing AO treatment guidelines in a prospective comparative cohort study. In addition to the follow up, featuring clinical documentation and registration of weight bearing milestones, new techniques for ambulatory measurements of loading (i.e. gait analysis by means of insoles) and non-invasive quantification of muscle mass will be used. This study will be performed in patients with (peri)- or intra-articular fractures of the pelvis/acetabulum and lower extremity after surgical treatment for which existing guidelines do not allow early full weight bearing in the first 6–12 weeks.

The primary research question is: How do the current guidelines (AO guidelines) and a permissive weight bearing protocol compare as to early recovery of functional outcome in trauma patients with fractures of the lower extremities after 6 months?

Secondary research questions are:

- To what extent is the use of a permissive weight bearing protocol for trauma patients with fractures of the lower extremities, as compared to treatment as usual (TAU), preferable in terms of costs, effects and utilities from both a hospital and a societal perspective?
- How do the current guidelines and a permissive weight bearing protocol compare as to complication rate in trauma patients with fractures of the lower extremities after 6 months?

### Hypothesis

#### Hypothesis 1

The permissive weight bearing protocol will lead to 1A: a better outcome at activity level (as measured with the Lower Extremity Functional Scale (LEFS)), 1B: a better early recovery at function level (as measured with the Brunnstrom Fugl-Meyer (BFM) test), 1C: a better participation (as measured with the SF-36) and 1D: a better quality of life (as measured with the EQ-5D-5L) in the first 6 months post-surgery, as compared to patients who are treated according to current standard guidelines. It is expected that in the long-term (i.e. 1 year) functional outcome will be similar between the treatment groups and will therefore not be the primary focus of this study [3]. We have chosen for the three scales mentioned above to cover the major outcome levels in the ICF model [17].

#### Hypothesis 2

The permissive weight bearing protocol for trauma patients with fractures of the lower extremities is more cost-effective compared to the non-weight bearing protocol and current guidelines.

#### Hypothesis 3

The rate of complications (e.g. failure of osteosynthesis, secondary displacement of fracture parts, non-union, infections) is equal or lower in patients who are treated according to the permissive weight bearing protocol compared to patients treated according to standard current guidelines.

## Methods/design

### Study design

**Fig. 1:**
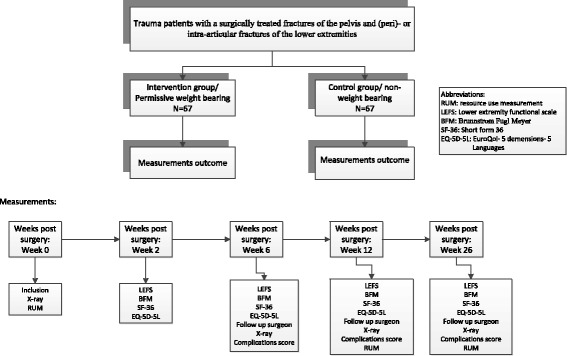
Flow chart of the study

This study is a prospective multicenter comparative cohort study in which two rehabilitation aftercare treatments will be contrasted, i.e. permissive weight bearing (PWB) and non-weight bearing (NWB) or the AO-guideline. The patient will be followed for 6 months (see Fig. 1). The inclusion time is 12 months.

Prior to participation, informed consent will be obtained from all participants.

This study has been approved by the Medical Ethics Committee of Maastricht University Medical Center, Maastricht, the Netherlands.

This study is registered in the Dutch Trial Register (NTR6077). Date of registration: 01–09-2016.

### Population

Trauma patients, aged 18 years or older, with a surgically treated fracture of the pelvis/acetabulum or a surgically treated (peri)- or intra-articular fracture of the lower extremities, where, according to the current guidelines, permissive weight bearing would not be allowed in the first weeks post-operatively, will participate.

Patients must be able to understand and follow instructions and written informed consent has to be obtained. Patients are screened for eligibility according to the inclusion and exclusion criteria presented in Table 1.

**Table 1:** Inclusion and exclusion criteria

Inclusion criteria	Exclusion criteria
• Trauma patients with surgically treated (peri)- or intra-articular fractures of the lower extremities, i.e. pelvic fractures, acetabular fractures, distal femur fractures, tibia plateau fractures, pilon fractures and foot fractures. According to the AO fracture classification type A, B, or C. • Age > = 18 years • Being able to understand the questionnaires and measurement instructions	• Restricted mobility due to other causes than the surgically treated (peri)- or intra-articular fracture, i.e. bilateral fractures, amputations, or ipsilateral significant fractures that hamper mobilization. • Severe non-fracture-related comorbidity of the lower extremity, e.g. congenital bone and tissue disorders. • No additional problem of rheumatic orthopaedic or neurological nature of the lower extremities (e.g. primary coxarthrosis or gonarthrosis) • Congenital bone and tissue disorders. • No informed consent

Participants for the intervention group, i.e. who will receive treatment according to the permissive weight bearing protocol, will be recruited in Maastricht University Medical Center (Maastricht) and in Zuyderland Medical Center (Heerlen). This intervention has already been implemented in these two hospitals, as well as in Adelante Rehabilitation Center (Hoensbroek).

The control group will be recruited in Viecuri Medical Center (Venlo), Maxima Medical Center (Veldhoven), Catharina Hospital (Eindhoven) and Elkerliek Hospital (Helmond). All participating centers are located in the Netherlands. The inclusion of the participants will start in October 2017 and will continue until September 2018.

### Randomization process

Randomization is not considered feasible because of the nature of the two different interventions. Implementation of these different protocols includes patient instructions as well as physical therapy guidance and nursing staff participation. A mix of protocols to be used on a ward is therefore considered not optimal because of information bias.

### Feasibility

From the trauma registration in all the six hospitals, every year more than 500 patients are admitted for surgical treatment of these specific lower extremity fractures that require unloaded aftercare according to the current guidelines (AO protocol). Accounting for an informed consent ratio of 50% and a lost-to-follow-up rate of 20%, an inclusion phase of one year will be necessary to complete this study.

### Sample size calculation

To date, no exact data on differences in functional outcome (LEFS) between the two different aftercare treatments for patients with fractures of the lower extremities are available. In two studies, the LEFS is expected to be 65 points (SD 10) in the non-weight bearing group [18, 19]. In this study we consider an increase in LEFS of 10% or more in the permissive weight bearing treatment group. In order to detect a difference between two independent means (two groups), assuming that an equal number of patients in both groups will be included, with an alpha of 5% and beta of 20% (power 80%), the sample size should be at least 51 for both groups, thus leading to a total number of 102. Anticipating a 20% drop out and 10% more patients to obtain equal groups, a total of 134 patients have to be recruited.

### Interventions

#### Permissive weight bearing

Instead of prescribing a rigorous regime of non-weight bearing, patients are instructed and trained to start weight bearing as tolerated. The limitation in weight bearing is only dependent on the patient’s perception of pain, feeling of instability, or redness and swelling at the site of the fracture. In therapy sessions the physiotherapists stimulate early weight bearing and instruct patients to perform exercises to achieve more weight bearing when tolerated. The goal of the protocol is not to achieve full weight bearing as quickly as possible, but rather to stimulate the patient to increase weight bearing depending on his ability to do so, while maintaining a safety margin to avoid complications or to detect complications early.

Non-weight bearing or restricted weight bearing (current guidelines or AO-guideline):

The standard aftercare treatment in surgically treated trauma patients with (peri)- or intra-articular fractures of the lower extremities is non-weight bearing or partial weight bearing [3]. According to the AO-protocol, postoperative management of (peri)- or intra-articular fractures of the lower extremities consists of non-weight bearing for 6–12 weeks followed by partial weight bearing with a 25% increase in weight loading every week [1]. Non-weight bearing is taught as per the institutional standard, based on the prescribed aftercare treatment. Insole pressure measurement system will be used to monitor the weight bearing and the compliance of the patients (see below).

### Data collection

Baseline measurements will be performed as soon as possible post-injury (= week 0). Further measurements will be taken at week 2, week 6, week 12 and week 26 post trauma (see also flow chart, Fig. 1).

### Demographic and medical variables

The following variables will be recorded:

- Gender
- Age
- Date and time of trauma
- Type of fracture
- Injury severity score (ISS)
- Type of operation
- Length of stay in hospital
- Complications before rehabilitation
- Weight bearing policy
- Time between accident and weight bearing
- Date of 100% weight bearing (insoles)
- Complications during rehabilitation.

### Primary outcome measures

ADL with Lower extremity functional scale (LEFS).

Outcome variables gauging functional outcome:

Score on LEFS [20] at 0, 2, 6, 12 and 26 weeks post-surgery.

LEFS: This is a questionnaire containing 20 questions about a person’s ability to perform everyday tasks. The LEFS can be used by clinicians as a measure of patients’ initial function, ongoing progress and outcome, as well as to set functional goals. The LEFS can be used to evaluate the functional impairment of a patient with a disorder of one or both lower extremities. It can be used to monitor the patient over time and to evaluate the effectiveness of an intervention. The questionnaire’s rating scale consists of 80 points. The lower the score the greater the disability [20].

### Secondary outcome measures

- Function (Brunnstorm Fugl-Meyer)
- Participation (SF-36)
- Improvement in quality of life (EQ-5D-5L)
- Reduction in health and society costs (RUM)
- Total complication rate

Outcome variables gauging functional outcome:

- Score on Brunnstrom Fugl-Meyer [21] at 0, 2, 6, 12 and 26 weeks post-surgery
- Score on SF-36 [22] at 0, 2, 6, 12 and 26 weeks post-surgery
- Score on EQ-5D-5L [23] at 0, 2, 6, 12 and 26 weeks post-surgery

Outcome variables gauging complication rate:

- Failure of the osteosynthesis, defined as loosening or breakage of implants
- Migration of fracture parts, defined as > 3 mm articular step-off and/or varus/valgus malalignment > 5 degrees
- Infection, defined as (but not limited to) purulent wound drainage, inflammation, erythema, fever, increased white blood cell (WBC) count, increased C-reactive protein (CRP) and/or increased erythrocyte sedimentation rate (ESR), necessitating admission for intravenous antimicrobial treatment and/or revision surgery
- Non-union, defined as no radiographic union achieved after 6 months or no progress in healing

Outcome variables gauging compliance [5, 6, 24–26]:

- Self-reported adherence to the fracture-related weight bearing protocol, will be recorded in medical records by physical therapists.
- Peak load (% body weight) and step duration (in seconds) as measured by the Sensistep [27] insole pressure measurement system. The Sensistep will be used by the patient only during daytime. The measurements with the Sensistep will continue until the patient has shown 100% weight bearing. The time for the latter to occur, may differ between patients.

Brunnstrom Fugl-Meyer: This is a test that evaluates the degree of synergy formation. The test consists of 55 test items that can be scored on an ordinal 3 - point scale (0–2 points). The total test consists of an examination of the upper extremity, an examination of the lower extremity and an examination of the balance. For our study we will only use the examination of the lower extremity. The maximum total score concerning the lower extremities is 34 points [21].

SF-36: This is a multi-purpose, short-form health survey with 36 questions. It yields an 8-scale profile of functional health and well-being scores, as well as psychometrics-based physical and mental health summary measures, and a preference-based health utility index. The higher the score the better the participation [22].

EuroQol: Both generic quality of life, as well as utilities, will be derived by means of the EQ-5D, which both will be administered to the patients. The EQ-5D is chosen because it is a widely used quality of life instrument (nationally and internationally) and it is recommended by the Dutch guidelines [28]. The EQ-5D contains 5 dimensions of health-related quality of life, namely mobility, self-care, daily activities, pain/discomfort and depression/anxiety. Each dimension can be rated at five levels ranging from ‘no problems’ to ‘major problems’.

Resource Use Measurement (RUM): for this study we will develop a RUM instrument especially designed for this group, based on existing questionnaires, which will measure all relevant costs aspects [29]. A resource use measure counts the frequency of defined health system resources; such as allowable charges, paid amounts, or standardized prices. Current approaches for measuring resource use range from broadly focused measures, such as per capita measures, which address total healthcare spending per person, to those with a more narrow focus, such as measures dealing with healthcare spending for an individual procedure.

Complication score:

Measurements will be taken at 6, 12, and 26 weeks post-surgery.

During scheduled visits to the physician, signs of osteosynthesis failure / infection / non-union / delayed union will be recorded in the study database.

Radiographic evaluation by a radiologist blinded for treatment allocation will be done at the same time intervals as the scheduled visits to the physician. Radiographs will be scored for signs of osteosynthesis failure / infection / non-union and migration of fracture parts, and results will be recorded in the study database.

### Statistical analyses

Data will be recorded in the digital study database and will be analyzed by a researcher blinded to the study groups the patients entered into.

Variations in case mix between centers can influence the interpretation of outcome data. Therefore, for each of the data sets collected, differences in outcome variable between the services will be tested using multiple MANCOVA’s, entering various indicators of case mix as co-variates (i.e. age, gender, ISS, number of complications, etc).

Insole pressures will be presented as mean ± SD. Statistical analysis of pressures involves repeated-measures two way ANCOVA using time as the within-group factor and treatment protocol as the between-group factor. Post-hoc group comparison at the different time points is only performed when the overall repeated-measures tests are statistically significant. A Bonferroni approach will be used to avoid spurious false positive findings. All scores will be tested for normality using the Shapiro-Wilk test. Multilevel factor analysis will be performed to determine independent factors related to the primary outcome parameter functional outcome.

Statistical analysis will be performed using the Statistical Package for Social Sciences (SPSS, IBM), version 23. Level of significance will be set at alpha< 0.05.

## Discussion

The main objective of this study is to examine the effectiveness and cost-effectiveness of a permissive weight bearing protocol for trauma patients with fractures of the lower extremities.

As there are no publications found about contrasts in (cost-) effectiveness between different aftercare protocols for the above mentioned patient group, it is important to investigate whether a permissive weight bearing protocol is more effective than the usual/current non-weight bearing protocol.

During the conceptualization of this study design an important choice had to be made concerning the randomization of the study. Randomization is not considered feasible because of the nature of the two different interventions. Implementation of these different protocols includes patient instructions as well as physical therapy guidance and nursing staff participation. A mix of treatment protocols on a ward is therefore considered not optimal because of information bias. However, one should take into account that not randomizing the study may introduce an observer bias, which may be a study limitation.

In the present study the definition of permissive weight bearing is as follows: Instead of prescribing a rigorous regime of non-weight bearing patients are instructed and trained to start bearing weight as tolerated. The limitation in weight bearing is only dependent on the patient’s perception of pain, feeling of instability, or redness and swelling at the site of the fracture. In therapy sessions the physiotherapists stimulate weight bearing and instruct patients to perform exercises to achieve more weight bearing if tolerated. The goal of the protocol is not to achieve full weight bearing as quickly as possible, but rather to stimulate the patient to increase weight bearing depending on his ability to do so, while maintaining a safety margin to avoid complications or to detect complications early.

Patient’s compliance with a non-weight bearing or limited weight bearing regime has been found to be poor [5, 6]. In this study, an insole pressure measurement system will be used to monitor the compliance of the patients.

To analyze specific complications; e.g. arthritis, the follow up period of 6 months is too short and could be a limitation in this study. To eliminate this limitation, a patient-questionnaire could be send to all patients after 2 years.

In conclusion, this paper describes the design of a prospective multicenter comparative cohort study that will investigate the (cost-) effectiveness of a permissive weight bearing aftercare protocol for trauma patients with fractures of the lower extremities. The inclusion of the patients will start in January 2018 and will continue until December 2018. The results of this study will give evidence whether a permissive weight bearing protocol is more effective than the current non-weight bearing protocol and thus should be introduced nation-wide.
